# AC-Bipolar Electropolymerization of 3,4-Ethylenedioxythiophene in Ionic Liquids

**DOI:** 10.1021/acs.langmuir.3c00120

**Published:** 2023-03-15

**Authors:** Zhenghao Chen, Yaqian Zhou, Elena Villani, Naoki Shida, Ikuyoshi Tomita, Shinsuke Inagi

**Affiliations:** † Department of Chemical Science and Engineering, School of Materials and Chemical Technology, Tokyo Institute of Technology, 4259 Nagatsuta-cho, Midori-ku, Yokohama 226-8502, Japan; ‡ College of Chemistry and Materials Science, Northwest University, Xi’an 710069, P. R. China; § Department of Chemistry and Life Science, Yokohama National University, 79-5 Tokiwadai, Hodogaya-ku, Yokohama 240-8501, Japan

## Abstract

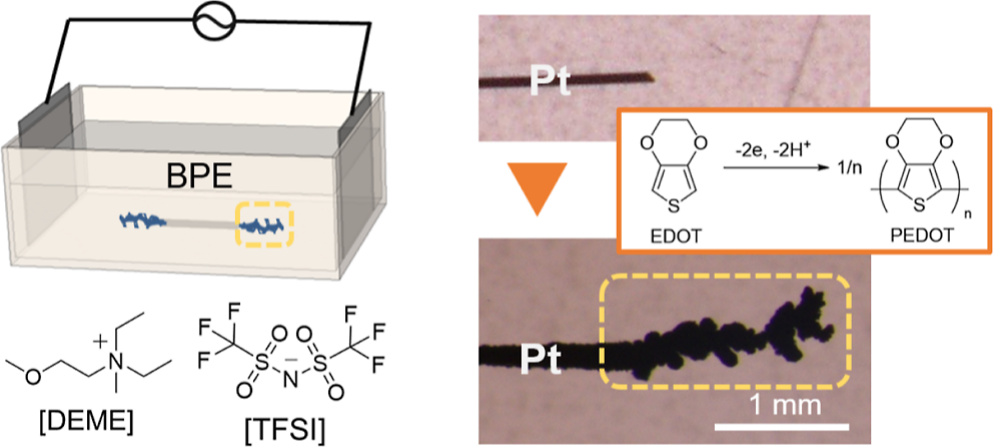

Recently, alternating current (AC)-bipolar electropolymerization of 3,4-ethylenedioxythiophene (EDOT) has been reported to produce poly(3,4-ethylenedioxythiophene) (PEDOT) fibers from the terminals of bipolar electrodes in acetonitrile solution (MeCN) containing low concentrations of supporting salts in a template-free manner. Here, we extend such methodology in ionic liquid (IL) media. Three kinds of ILs, diethylmethyl(2-methoxyethyl)ammonium tetrafluoroborate ([DEME][BF_4_]), 1-ethyl-3-methylimidazolium tetrafluoroborate ([EMIM][BF_4_]), and diethylmethyl(2-methoxyethyl)ammonium bis(trifluoromethylsulfonyl)imide ([DEME][TFSI]), with different electric field transmission efficiencies and diffusion coefficients were employed as solvents for the AC-bipolar electropolymerization of EDOT. A variety of PEDOT morphologies were obtained in these three ILs, showing a relationship with the physicochemical properties of the ILs. We successfully confirmed the growth of PEDOT fibers in ILs and systematically discussed the factors that influenced their growth.

## Introduction

**Figure 1:**
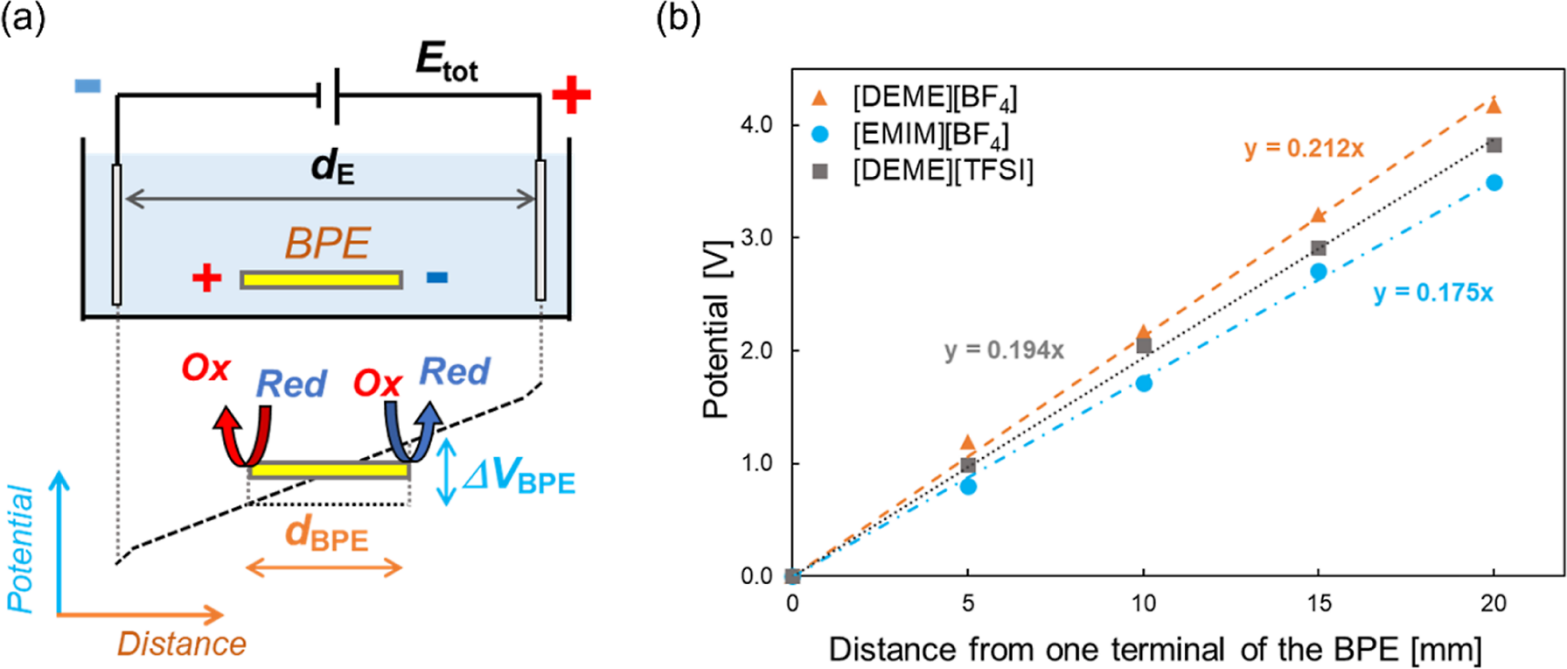
(a) Schematic illustration of the DC-bipolar electrochemical system. AC-bipolar electrochemistry follows the same principle. (b) Potential distribution in the three ILs under the application of an AC external electric field (10 V, 5 Hz, SQU) measured with the setup shown in Figure S1.

Bipolar electrochemistry is a green and unique approach for the synthesis of organic, inorganic, and polymeric materials due to the requirement of only a small amount of electrolyte and the generation of electrochemical reactions on a bipolar electrode (BPE) even in the absence of a direct Ohmic contact.^1−4^ Therefore, this electrochemical technique can be exploited in many interesting applications such as sensing devices,^5^ electric field-driven bioinspired machines,^6^ wireless modifications of conductive objects,^7^ and gradient material synthesis.^8^ Generally, in a bipolar electrochemical system, a pair of driving electrodes connected to an external power source generates a uniform electric field across the bulk solution with the use of a low concentration of electrolyte. When a conducting material is placed in the bulk solution, a potential difference between its terminals can induce electrochemical reactions on the conductor, i.e., the BPE, in a wireless manner (Figure 1a). The potential difference between the two ends of the BPE (Δ*V*_BPE_) can be simply estimated with the use of two probes set upon the BPE during the application of a certain voltage (*E*_tot_) to the driving electrodes. Moreover, electrochemical reactions tend to occur at the opposite terminals of BPEs, where the largest Δ*V*_BPE_ is generated according to the principle of bipolar electrochemistry. An important parameter to be considered for the choice of the electrolyte, aiming to generate a suitable electric field for reactions, is the electric field transmission efficiency, EFTE (θ),^9^ which is the ratio between the electric field intensity inside the cell (ε_eff_) and the applied electric field intensity between the driving electrodes (ε) (see the Experimental Section).

Bradley, Gogotsi, and co-workers reported that carbon nanotubes (CNTs) can work as BPEs to achieve the electropolymerization of pyrrole, which formed the corresponding polypyrrole (PPy) deposition at one terminal of the CNTs under the application of direct current (DC) voltage.^10^ Loget, Kuhn, and co-workers also reported the DC-bipolar electropolymerization of pyrrole on one end of a carbon tube used as a BPE and the simultaneous metal electrodeposition at the other end of the BPE.^11^ Inspired by these reports, our group applied alternating current (AC) voltage with the square wave (SQU) for bipolar electropolymerization applications. Note that AC-bipolar electrochemistry follows the same principle of DC-bipolar electrochemistry (Figure 1a) because it just switches between constant DC-voltages. When gold (Au) wires were used as BPEs in a bipolar electrolytic system under conditions of the AC voltage, the electropolymerization of 3,4-ethylenedioxythiophene (EDOT) proceeded at both ends of Au wires,^12−14^ giving the corresponding conducting polymer depositions with the morphologies of fibers^15−18^ and films.^19,20^ According to the fact that an external electric field drives the generation of redox reactions on BPEs in the electrolyte containing a low concentration of supporting salts, electrogenerated oligomers/polymers possessing cationic charges derived from concurrent doping undergo electrophoresis under the influence of the electric field. This synergetic effect of electrophoresis and electrolytic reaction affords the fabrication of anisotropic conducting polymer materials without the use of templates.^13^

The use of ionic liquids (ILs)^21,22^ as green solvents has received considerable attention in organic electrosynthesis,^23^ electrodeposition of metals,^24^ electrochemical devices,^25^ and energy applications,^26^ due to their non-flammable, non-volatile, and recyclable properties, as well as the considerably wider potential window compared to other common solvents. Furthermore, they can also be disposed of in a safer manner compared to many other liquids after electrochemical reactions. Fuchigami and co-workers reported the electropolymerization of pyrrole in the 1-ethyl-3-methylimidazolium trifluoromethanesulfonate ([EMI][CF_3_SO_3_]) IL with the conventional three-electrode system, demonstrating that the polymerization rate, electrochemical capacity, and electroconductivity of the obtained PPy could be well improved.^27^ On the other hand, Zigah and co-workers employed 1-butyl-3-methylimidazolium bis(trifluoromethylsulfonyl)imide ([BMIM][TFSI]) IL as the electrolyte for the electropolymerization of pyrrole using the bipolar electrolytic system.^28^ In this case, when an Au plate was used as a BPE under the application of a DC voltage, the PPy film deposited at one side of the Au plate shows a thinner and smoother morphology than the one prepared in acetonitrile. To the best of our knowledge, this report is the only one concerning the use of ILs as solvents for bipolar electrochemistry experiments, demonstrating that BPEs can be driven even in highly concentrated electrolytes and a sufficient potential difference can be generated in the bulk solution as well. This finding can open the door to a new stage of bipolar electrochemistry that takes advantage of ILs.

In this context, we report the AC-bipolar electropolymerization of the EDOT monomer in ILs and the relative investigation of the polymerization behavior and resulting morphologies of the corresponding polymer deposits. Three ILs, diethylmethyl(2-methoxyethyl)ammonium tetrafluoroborate ([DEME][BF_4_]), 1-ethyl-3-methylimidazolium tetrafluoroborate ([EMIM][BF_4_]), and diethylmethyl(2-methoxyethyl)ammonium bis(trifluoromethylsulfonyl)imide ([DEME][TFSI]), were employed for the investigation. Electrochemical measurements in ILs were initially performed to construct the bipolar electrolytic system. Regarding the AC-electropolymerization process, the propagation of poly(3,4-ethylenedioxythiophene) (PEDOT) fibers was successfully observed at the end of a platinum (Pt) BPE wire in the case of [DEME][TFSI], while PEDOT clusters were deposited at the terminals of the BPE in [DEME][BF_4_], and the deposition of a film with grains was observed in [EMIM][BF_4_]. The different morphologies and deposition modes of PEDOT fibers were also discussed in detail in terms of the EFTE value and the diffusion coefficient of substrates in the ILs. These studies clarified that ILs are available as electrolytic media for the AC-bipolar electropolymerization of EDOT, providing PEDOT with different growth behaviors depending on the properties of the IL.

## Experimental Section

### Materials

EDOT, 1,4-benzoquinone (BQ), diethylmethyl(2-methoxyethyl)ammonium tetrafluoroborate ([DEME][BF_4_]), 1-ethyl-3-methylimidazolium tetrafluoroborate ([EMIM][BF_4_]), diethylmethyl(2-methoxyethyl)ammonium bis(trifluoromethylsulfonyl)imide ([DEME][TFSI]), and tetrabutylammonium perchlorate (Bu_4_NClO_4_) were purchased from commercial sources and used without further purification. Platinum (Pt) wires and plates were obtained from the Nilaco corporation.

### Instruments

Bipolar electrolysis was performed by feeding a constant voltage from an EC1000SA AC/DC power source (NF Corporation) to the driving electrodes. Scanning electron microscopy (SEM) observations were performed using a JEOL JSM-6610LA microscope. The viscosity of ILs was measured with a Japan A&D Company SV-10A viscometer. The conductivity of a dilute solution was measured with a HORIBA LAQUA conductivity cell (submersible type) 3552-10D. Linear sweep voltammetry measurements were performed on an ALS model 2325 potentiostat. The potential distributions of the AC electric field were measured by the SANWA PC773 digital multimeter.

### Cell Configuration

**Figure 2:**
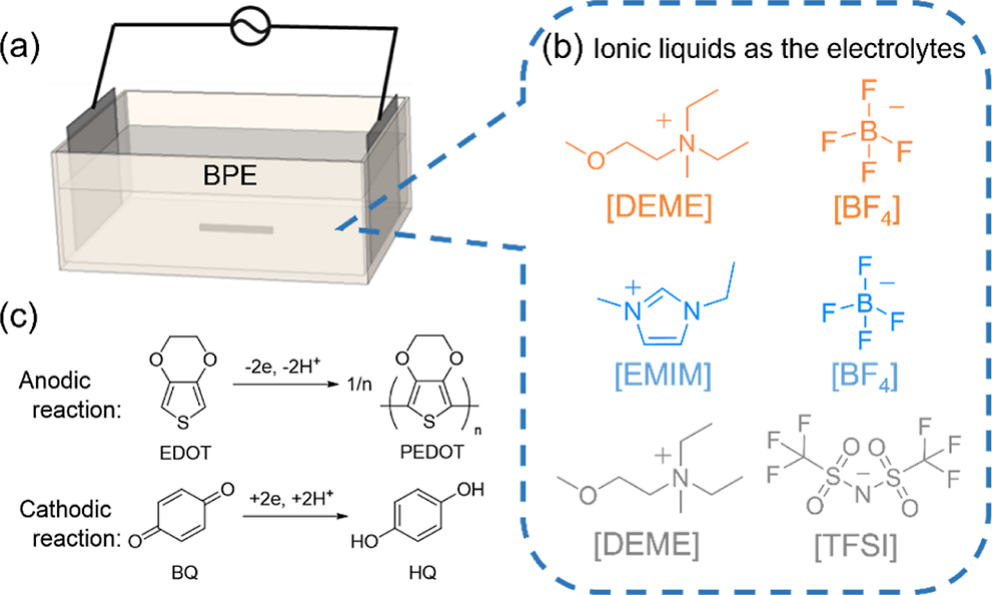
Concept of the electrolytic system: (a) schematic illustration of the electrolytic cell containing (b) different ILs as electrolytes and (c) simultaneous anodic oxidation reaction of EDOT to produce PEDOT and cathodic reduction reaction of 1,4-benzoquinone (BQ) to generate 1,4-hydroquinone (HQ).

The electrolytic cell was made of polypropylene (40 mm × 30 mm × 15 mm), equipped with two Pt driving electrodes (20 mm × 20 mm, distance: 40 mm) and a Pt wire (ϕ = 50 μm, 15–20 mm in length) as the BPE placed between the driving electrodes. An AC power supply was externally connected to the pair of driving electrodes (Figure 2a).

### Estimation of Δ*V*_BPE_

Under the application of a cell voltage (*E*_tot_) to the driving electrodes, two Pt probes connected with a voltmeter were set upon the surface of the BPE, where the solution potential difference between the two probes (Δ*V*_BPE_) was measured (Figure S1).

### Estimation of EFTE

According to Figure S1, the solution potential difference linearly changed around the BPE, indicating that a constant intensity of the electric field was generated in each IL. The electric field transmission efficiency, EFTE (θ), i.e., the ratio between the electric field intensity around the BPE (ε_eff_ = Δ*V*_BPE_/*d*_BPE_) and the applied electric field intensity between the driving electrodes (ε = *E*_tot_/*d*_E_), was estimated according to eq 1, where *d*_E_ is the distance between the driving electrodes and *d*_BPE_ is the length of the BPE
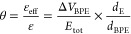
1

### Determination of the Length of the PEDOT Fibers

The PEDOT fibers obtained by AC-bipolar electropolymerization were transferred onto a carbon tape after synthesis. After careful washing with MeCN and drying, the fiber length was measured from the SEM images. The lengths of four fibers obtained from one experiment were determined, and the average value was calculated.

## Results and Discussion

Figure 2a shows the bipolar electrolytic setup composed of a polypropylene cell equipped with a pair of Pt driving electrodes connected to a power source, a Pt wire (ϕ = 50 μm) as a BPE, and IL as the electrolyte containing 50 mM EDOT and 10 mM 1,4-benzoquinone (BQ). The compositions and chemical structures of the three ILs, i.e., [DEME][BF_4_], [EMIM][BF_4_], and [DEME][TFSI] are presented in Figure 2b. The potential distribution in each IL during the application of AC-voltages between the driving electrodes was directly measured by using two probes connected with a voltmeter, demonstrating the formation of a gradient profile in all three ILs, as shown in Figure 1b. The linear sweep voltammograms of ILs showed their wide potential windows, demonstrating their suitability as inert electrolytic media (Figure S2). Based on the principle of bipolar electrochemistry, to trigger the simultaneous oxidation of EDOT and the sacrificial reduction of BQ at the opposite poles of the BPE (Figure 2c), the sum of the anodic and cathodic overpotentials (Δ*V*_BPE_) must be larger than their onset potential difference (Δ*V*_min_). The Δ*V*_min_ values were measured in each IL by linear sweep voltammetry, as shown in Figures S3–S5. When the oxidation of EDOT proceeds on the anodic side of the BPE, the sacrificial reduction of BQ to give 1,4-hydroquinone (HQ) occurs on the cathodic side simultaneously. Therefore, it is expected that the PEDOT deposition will be obtained at both ends of the BPE under the AC-bipolar electrochemical condition.

**Figure 3:**
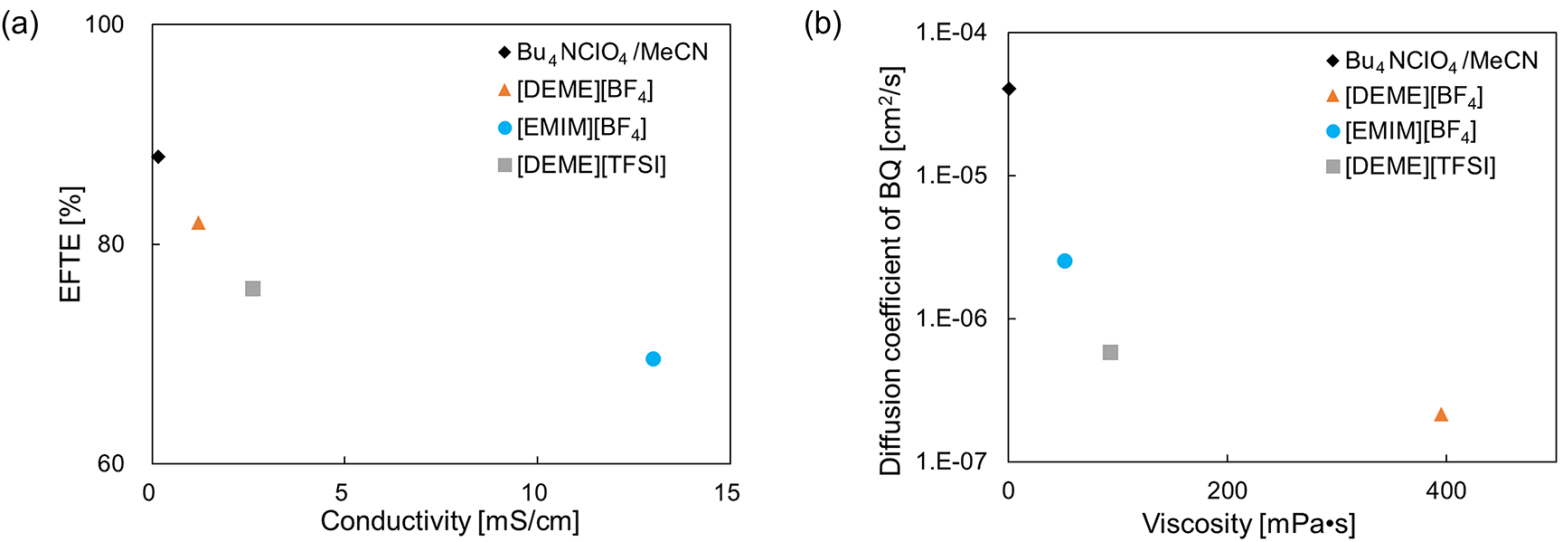
Scattered plots of (a) EFTE vs conductivity and (b) diffusion coefficient of BQ vs viscosity.

First, to explore the conditions for bipolar electrolysis of the target redox reactions in the three ILs, the EFTE value and diffusion coefficient of BQ in each IL were estimated at the constant temperature of 25 °C to keep the viscosity stable (viscosity curves are shown in Figure S6). These results and the ionic conductivity of each IL are summarized in Table 1. The diffusion coefficients of molecules exhibiting a reversible redox couple can be determined by cyclic voltammetry. Since the cyclic voltammogram of EDOT showed an irreversible oxidation wave, the diffusion coefficients were calculated from the cyclic voltammogram of BQ (Figures S7 and S8) to discuss the differences in mass transport of small organic molecules in ILs. For comparison with our previous studies on AC-bipolar electropolymerization in a dilute electrolytic solution,^15^ the properties of 1 mM Bu_4_NClO_4_ acetonitrile solution were also included. As shown in Table 1, the [DEME][BF_4_] IL had the highest viscosity (395 mPa·s) and the lowest conductivity (1.2 mS/cm) among the three ILs. On the other hand, the [EMIM][BF_4_] IL showed the lowest viscosity (51 mPa·s) and the highest conductivity (14 mS/cm). The viscosity and conductivity of [DEME][TFSI] were middle values among the three ILs. The key relationships between EFTE and conductivity and between the diffusion coefficient and viscosity are displayed in Figure 3a,b, respectively. Since a large EFTE value is advantageous for driving redox reactions on BPEs, [DEME][BF_4_] and [DEME][TFSI] are theoretically suitable for the bipolar electrochemical system. As for the diffusion coefficient, ILs with low viscosity such as [EMIM][BF_4_] and [DEME][TFSI] are expected to show better mass transfer and a faster reaction rate. Although the Bu_4_NClO_4_/acetonitrile dilute solution showed the largest EFTE and diffusion coefficient values, these ILs were found to be potential candidates as a medium for the bipolar electrochemical system.

**Table 1:** Physicochemical Properties of MeCN-Based Dilute Solution and ILs at 25 °C

electrolyte	concentration of electrolyte or IL [mM]	viscosity[mPa·s]	conductivity[mS/cm]^a^	EFTE [%]	Δ*V*_min_ [V]^b^	diffusion coefficient (*D*_O_) of BQ in electrolyte solution [cm^2^/s]
Bu_4_NClO_4_	1	<0.4	0.16	88.1	1.82	4.02 × 10^–^^5^^c^
[DEME][BF_4_]	5063	395	1.2	84.8	1.27	2.15 × 10^–^^7^
[EMIM][BF_4_]	6516	51	14	70.0	1.40	2.55 × 10^–^^6^
[DEME][TFSI]	3330	93	2.6	77.6	1.44	5.86 × 10^–^^7^

a The conductivity of Bu_4_NClO_4_/MeCN solution was measured with the conductivity cell. The conductivity data of ILs were obtained from the product information supplied by the commercial source.

b Δ*V*_min_ is the minimum potential difference required to induce redox reactions on the BPE (onset potential difference of EDOT and BQ).

c Estimated by the voltammogram measured in 10 mM of electrolyte because an accurate linear approximation could not be obtained with the voltammogram in 1 mM solution.

**Figure 4:**
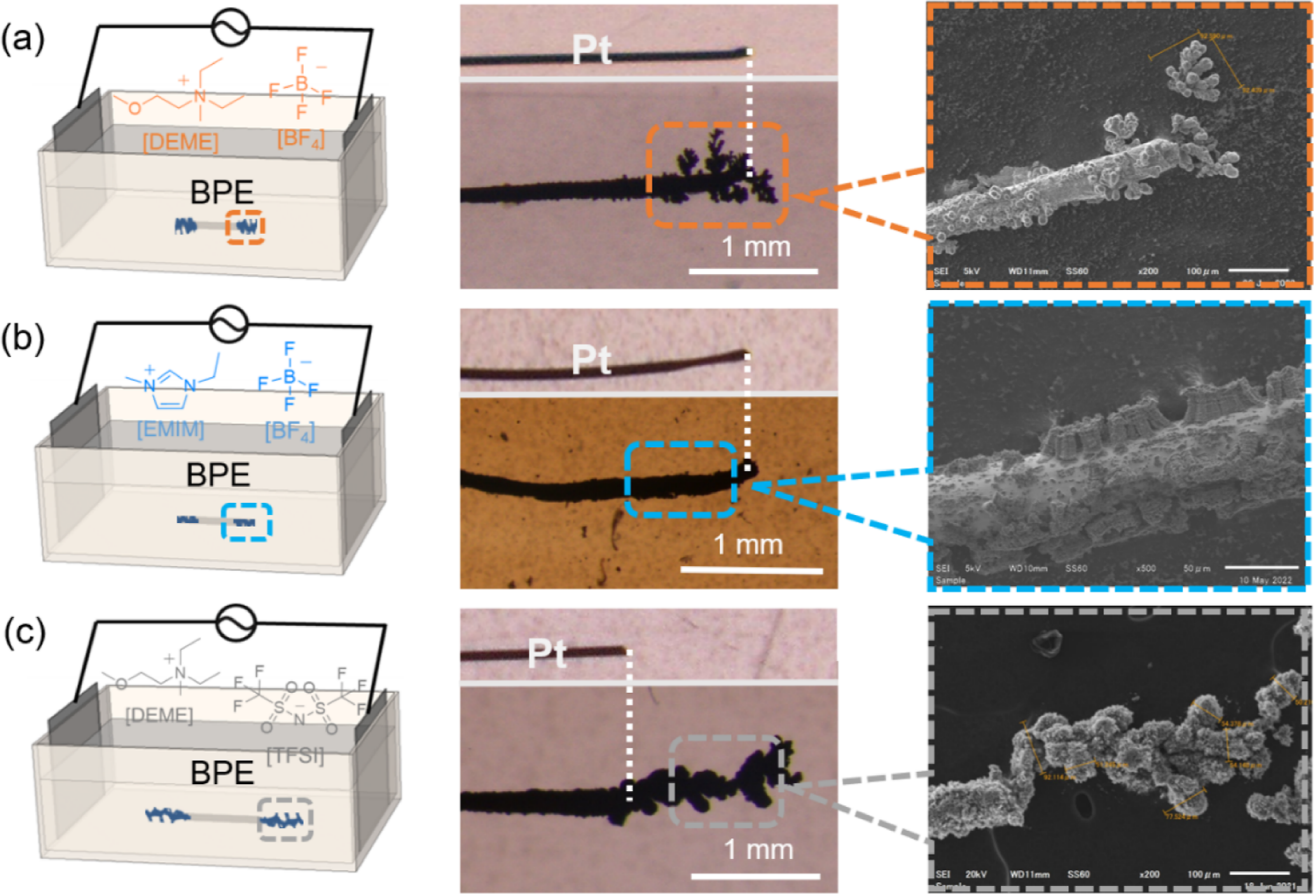
AC-bipolar electropolymerization of EDOT in the three ILs and the optical microscopy and SEM images of the obtained PEDOT deposits. (a) Branched PEDOT clusters in [DEME][BF_4_], (b) the PEDOT film covering the BPE in [EMIM][BF_4_], and (c) the linear PEDOT fiber in [DEME][TFSI].

Successively, the AC-bipolar electropolymerization of EDOT was investigated using a Pt wire (ϕ = 50 μm) as a BPE in the three ILs as shown in Figure 4. When an optimized AC voltage of 10 V (5.0 Hz, SQU) was applied to the driving electrodes, the Δ*V*_BPE_ value was estimated considering the EFTE and *d*_BPE_ values in each IL. Redox reactions were generated on the BPE in all three ILs, where the anodic electropolymerization of EDOT and reduction of BQ proceeded simultaneously at both terminals of the BPE to produce PEDOT deposits. No undesired electropolymerization was observed at the driving electrodes (Figure S9). During the optimization of electrochemical parameters, we changed the frequency and applied voltage (Figures S10 and S11). At a lower frequency (1 Hz), the conducting polymer deposition on the end of BPE became disordered, and some deposition diffused into the solution. On the other hand, the higher frequency (50 Hz) resulted in film formation covering the BPE wires (Figure S10). When a higher voltage (20 V) was applied to the driving electrodes, bubbles were generated at BPEs, presumably due to the electrolysis of contaminating water or the decomposition of ILs. Moreover, the electropolymerization of PEDOT at the driving electrodes proceeded intensely (Figure S11). After 2 h of electrolysis (10 V, 5 Hz, SQU), the PEDOT deposits with morphologies of thin films and fibers were observed with optical microscopy and SEM (Figure 4).

Theoretically, [DEME][BF_4_] is the most suitable IL for the bipolar electrochemical system according to its high EFTE value and the estimated Δ*V*_BPE_ of ca. 4.24 V (*d*_BPE_ = 20 mm), which is large enough to promote electrochemical reactions at the BPE terminals. Indeed, short and branched clusters were observed at the ends of the Pt BPE (Figure 4a). However, the extremely high viscosity of this IL negatively influenced the mass transport hampering the electrodeposition process^29^ and, at the same time, also affected the electrophoretic motion of cationic oligomers/polymers, with the result to prevent fine fiber formation, as we previously reported for a dilute electrolyte solution.

In the case of [EMIM][BF_4_] IL, which showed the lowest viscosity and a Δ*V*_BPE_ value of ca. 2.63 V (*d*_BPE_ = 15 mm), the obtained PEDOT deposit was not of the fiber form but rather a film that covered the surface of the BPE over 1 mm in length from its terminal (Figure 4b). Although the highest diffusion coefficient of redox species in this IL was beneficial for the reaction rate, the small electric field intensity, due to the low EFTE value, seemed not sufficient to induce the anisotropic growth of the PEDOT fiber.

Finally, [DEME][TFSI] with a Δ*V*_BPE_ value of ca. 3.88 V (*d*_BPE_ = 20 mm) and a middle value of both EFTE and diffusion coefficient among the three ILs, gave the PEDOT fibers linear morphology (Figure 4c). The diameter of the fiber was estimated from SEM analysis to be ca. 50–80 μm (the fiber length was 1–2 mm), which was significantly shorter and thicker than the PEDOT fibers obtained in the dilute solutions.^15,16,18^ Although the physicochemical properties of [DEME][TFSI] are well balanced and similar to those of the dilute electrolyte solution, the high viscosity and high ion concentration of this IL presumably decreased the electrophoretic effect of the cationic oligomers/polymers, hindering long fiber growth.

In our previous study, we could control the degree of branching of the PEDOT fibers by physically limiting the monomer diffusion toward the BPE using a micro-space around the BPE.^16^ Hence, in the bipolar electrochemical system using highly viscous ILs, we also recognized that the diffusion coefficient of the monomer is a key factor for the deposition process. Based on this assumption, [DEME][TFSI], which had the relatively low diffusion coefficient among the three ILs investigated, limited the monomer supply to the lateral side of the BPE. On the other hand, the moderate EFTE value could assist the electrophoresis of cationic oligomers/polymers, resulting in the anisotropic growth of PEDOT, which enabled the monomer supply to the frontier of the fiber in a similar manner to the previous case using a micro-space in a dilute electrolyte solution.

## Conclusions

In conclusion, AC-bipolar electropolymerization of EDOT in three ILs, [DEME][BF_4_], [EMIM][BF_4_], and [DEME][TFSI], was successfully demonstrated, which produced PEDOT clusters, films, and fibers having different morphologies. The difference in morphologies of the PEDOT deposition mode was also discussed in detail in terms of physicochemical properties of the three ILs, such as EFTE and the diffusion coefficient of the substrates. In particular, [DEME][TFSI], having middle EFTE and diffusion coefficient values among the three ILs, gave the PEDOT fibers linear morphology in a similar manner to the previous report using a dilute electrolyte solution. These studies clarified that ILs are suitable as electrolytic media for AC-bipolar electropolymerization even in their high ionic concentrations. Furthermore, ILs such as [DEME][TFSI] can be recovered by a simple purification process and thus reused without significant changes in their properties. Considering their features such as non-volatility and non-flammability, the use of ILs is an ideal approach to realize environmentally friendly electrosynthesis.

## Supporting Information Available

Estimation of Δ*V*_BPE_ and ETFE values; voltammograms of ILs and of EDOT and BQ in ILs; viscosity experiment of ILs; measurement of the diffusion coefficient; and electrolysis results at different frequencies and voltages (PDF)
