# Supplementary material for: AC-Bipolar Electropolymerization of 3,4-Ethylenedioxythiophene in Ionic Liquids
Source: Langmuir. 2023 Mar 15;39(12):4450–5. doi: 10.1021/acs.langmuir.3c00120 (PMC10061915; doi:10.1021/acs.langmuir.3c00120)
Supplement: Supplementary file 1 — la3c00120_si_001.pdf [file la3c00120_si_001.pdf]

Supporting Information

AC-Bipolar Electropolymerization of 3,4-Ethylenedioxythiophene in Ionic Liquids

Zhenghao Chen,[a] Yaqian Zhou,[b] Elena Villani,[a] Naoki Shida,[c] Ikuyoshi Tomita,[a] and Shinsuke Inagi[a]*

a. Department of Chemical Science and Engineering, School of Materials and Chemical Technology, Tokyo Institute of Technology, 4259 Nagatsuta-cho, Midori-ku, Yokohama 226-8502, Japan
b. College of Chemistry and Materials Science, Northwest University, Xi'an 710069, P. R. China
c. Department of Chemistry and Life Science, Yokohama National University, 79-5 Tokiwadai, Hodogaya-ku, Yokohama 240-8501, Japan

* Corresponding author: inagi@cap.mac.titech.ac.jp (S. I.)

TABLE OF CONTENTS

Estimation of the potential difference between the terminals of BPEs (ΔV_{BPE})………………..S3

The electric field transmission efficiency (EFTE)……………………………………………...S4

Potential window of the three ILs………………………………………………………………S5

Onset potential analysis for EDOT and BQ in ILs……………………………………………...S6

Viscosity curves of ILs………………………………………………………………………….S8

Measurement of the diffusion coefficient of 1,4-benzoquinone (BQ) in each IL………………S9

AC-bipolar electropolymerization for 2 h at 10 V with 5 Hz (SQU)………………………….S12

AC-bipolar electropolymerization for 2 h at 10 V with 1 Hz and 50 Hz (SQU)………………S13

AC-bipolar electropolymerization for 1 h at 20 V with 5 Hz (SQU)……………...……….....S14

References……………………………………………………………………………………..S15

Estimation of the potential difference between the terminals of BPEs (ΔV_{BPE})

The solution potential differences around the BPE in the electrolytic cell were directly measured by a voltmeter using two tip probes, where one tip (the blue points in Figure S1) was fixed on the surface of one terminal of the BPE (distance = 0 mm) and the other tip (the red points in Figure S1) was placed at different distances from the fixed tip. Average values were taken from the two lines of potential distribution data and plotted in Figure 1b.

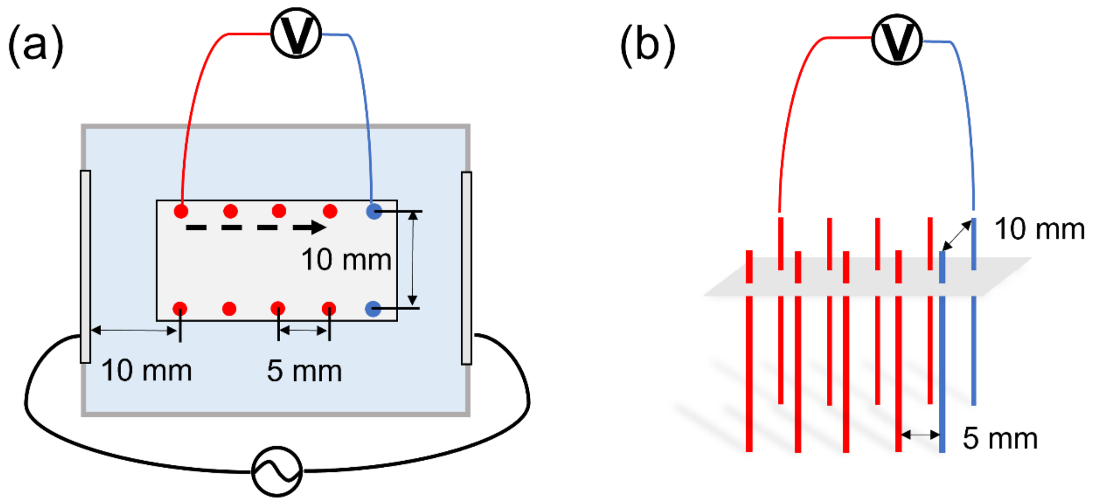

Figure S1. (a) Top view of the measurement setup for the potential distribution around the BPE. (b) Side view of the probes that were fixed at the insulating board to keep the distance from each other constant.

The electric field transmission efficiency (EFTE)

The electric field transmission efficiency[1], EFTE (θ), i.e., the ratio between the electric field intensity around the BPE ($\varepsilon_{eff} = \Delta V_{BPE}/d_{BPE}$) and the applied electric field intensity between the driving electrodes ($\varepsilon = E_{tot}/d_E$), was estimated according to Eq. 1, where d_E is the distance between the driving electrodes and d_{BPE} is the length of the BPE:

$$\theta = \frac{\varepsilon_{\mathrm{eff}}}{\varepsilon} = \frac{\Delta V_{BPE}}{E_{\mathrm{tot}}} \times \frac{d_{\mathrm{E}}}{d_{\mathrm{BPE}}} \qquad (1)$$

Here, E_{tot} = 10 V (5 Hz, SQU) was applied to the driving electrodes in an electrolytic cell (40 mm × 30 mm × 15 mm); therefore, ε can be calculated as 0.25 V/mm. The ε_{eff} values in ILs correspond to the slope of each potential distribution curve in Figure 1b. The estimated EFTE values are summarized in Table S1.

Table S1. Measurement of ε_{eff} and ε values.

IL	ε_{eff} [V/mm]	ε [V/mm]	θ [%]
[DEME][BF_4]	0.212	0.25	84.8
[EMIM][BF_4]	0.175	0.25	70.0
[DEME][TFSI]	0.194	0.25	77.6

Potential window of the three ILs

Linear sweep voltammetry measurements were carried out in [DEME][TFSI], [EMIM][BF_4] and [DEME][BF_4] using a Pt disk working electrode (ϕ = 3 mm), a Pt counter electrode (20 mm × 20 mm) and a saturated calomel electrode (SCE) as a reference electrode at scan rates of 100 mV/s.

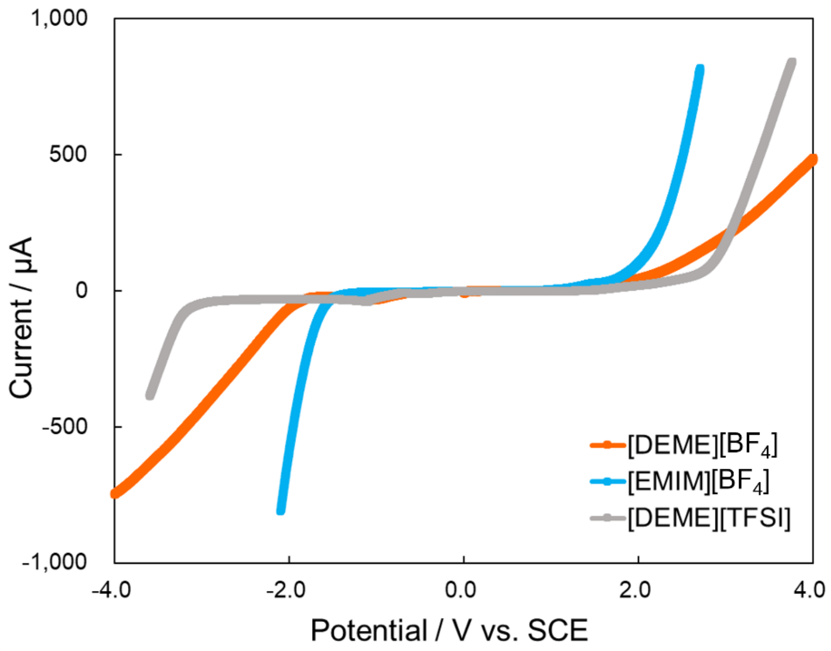

Figure S2. Potential windows of the three ILs.

Onset potential analysis for EDOT and BQ in ILs

Linear sweep voltammetry measurements of EDOT and BQ were carried out in ILs [DEME][BF_4], [EMIM][BF_4] and [DEME][TFSI] (Figures S3–S5) to assess their onset potentials for oxidation and reduction reactions, respectively.

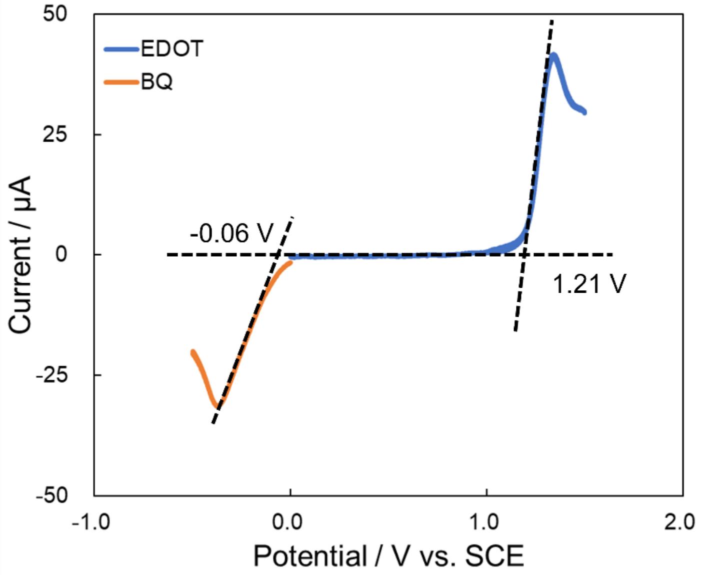

Figure S3. Linear sweep voltammograms of EDOT (10 mM) and BQ (10 mM) measured independently in [DEME][BF_4] using a Pt disk working electrode (ϕ = 3 mm) and a Pt counter electrode (10 mm × 10 mm) at scan rates of 100 mV/s.

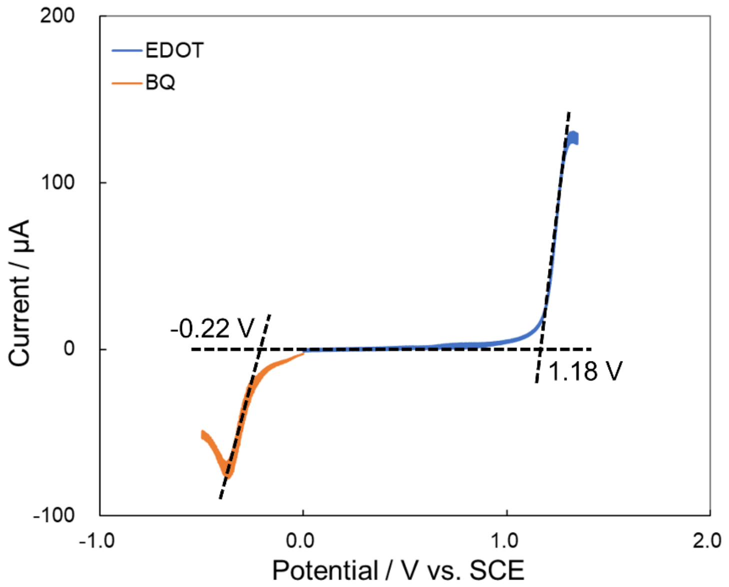

Figure S4. Linear sweep voltammograms of EDOT (10 mM) and BQ (10 mM) measured independently in [EMIM][BF_4] using a Pt disk working electrode (ϕ = 3 mm) and a Pt counter electrode (10 mm × 10 mm) at scan rates of 100 mV/s.

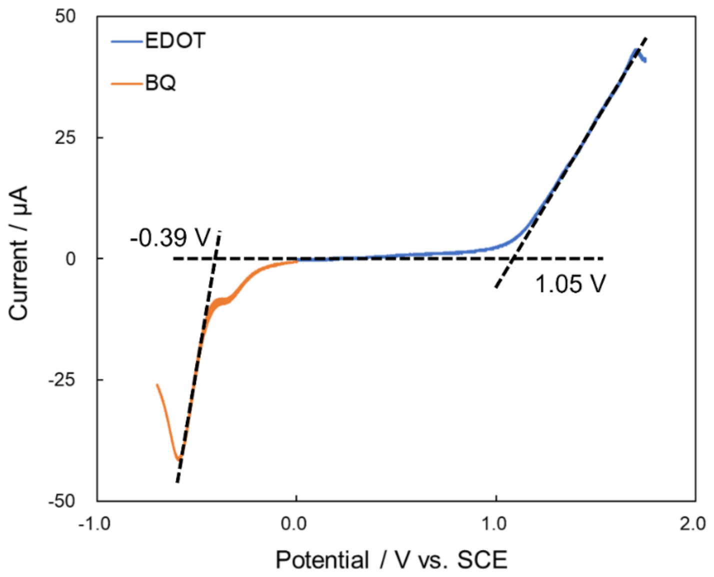

Figure S5. Linear sweep voltammograms of EDOT (10 mM) and BQ (10 mM) measured independently in [DEME][TFSI] using a Pt disk working electrode (ϕ = 3 mm) and a Pt counter electrode (10 mm × 10 mm) at scan rates of 100 mV/s.

Viscosity curves of ILs

The viscosity of the ILs was measured with a Japan A&D Company SV-10A viscometer, and the cell temperature was controlled with an EYELA NCB1210 chiller. All viscosity data were collected with the Rs-Visco software from Japan A&D Company.

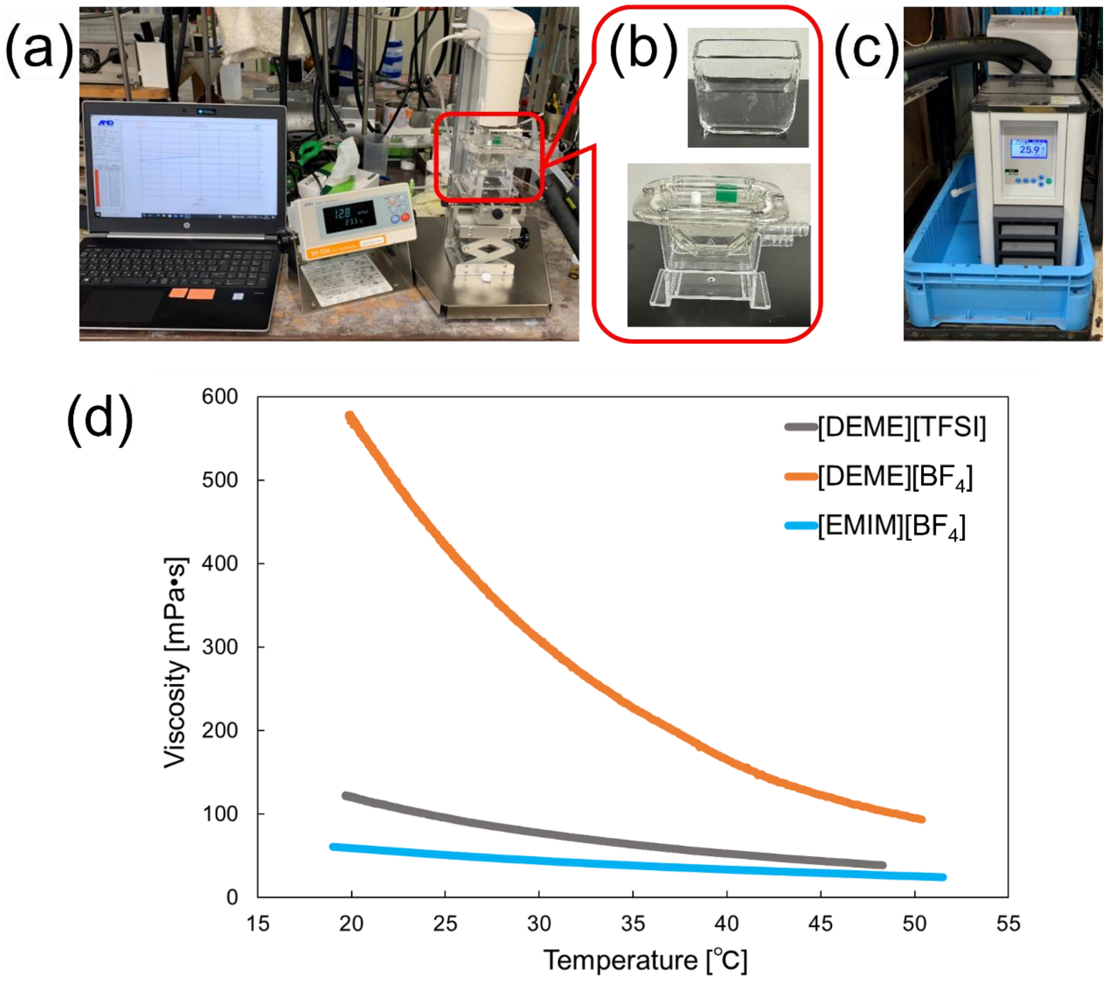

Figure S6. (a) The viscosity measurement system composed of a viscometer and a laptop for data collection. (b) 15 mL of IL were added to the glass cell, which was set to the plastic heat conduction mold. (c) The temperature controlling device. (d) Viscosity curves of the three ILs as a function of the temperature (the ILs were heated at ~ 50 °C and then cooled down at room temperature).

Measurement of the diffusion coefficient of 1,4-benzoquinone (BQ) in each IL

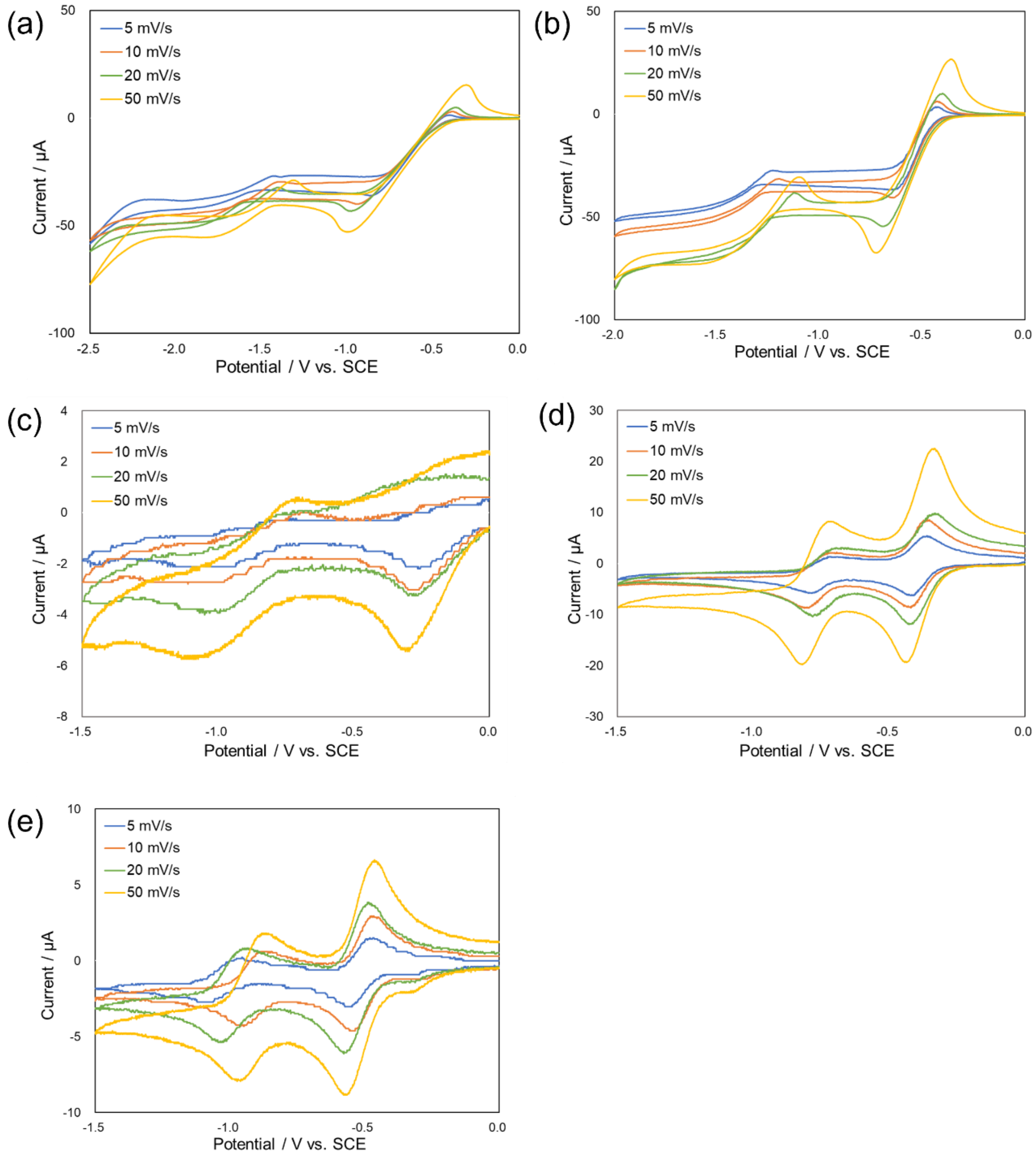

Figure S7. Cyclic voltammograms of BQ (10 mM) obtained with a Pt disk working electrode (ϕ = 1.6 mm) and a Pt counter electrode (10 mm × 10 mm) at scan rates of 5, 10, 20 and 50 mV/s in (a) 1 mM Bu_4NClO_4 acetonitrile solution, (b) 10 mM Bu_4NClO_4 acetonitrile solution, (c) [DEME][BF_4] IL, (d) [EMIM][BF_4] IL and (e) [DEME][TFSI] IL.

For the determination of the diffusion coefficient (D_O) of BQ from the voltammograms reported in Figure S7, the following equation is considered:[2]

$$i_p{}^c = 0.4463\left(\frac{F^3}{RT}\right)^{1/2} n^{3/2}AD_O^{1/2}C_O^*v^{1/2} \qquad (2)$$

where $i_p{}^c$ in amperes is the cathodic peak current, n is the number of electrons exchanged in the redox process, A in cm^2 is the area of the working electrode, C_O^* in mol/L is the concentration of BQ in the bulk solution and v in V/s is the scan rate.

When T = 298 K, the peak current $i_p{}^c$ changes to:

$$i_p{}^c = (2.69 \times 10^5)n^{3/2}AD_O^{1/2}C_O^*v^{1/2} \qquad (3)$$

It follows that:

$$\frac{i_p{}^c}{v^{1/2}} = (2.69 \times 10^5)n^{3/2}AD_O^{1/2}C_O^* \qquad (4)$$

since $k = i_p{}^c/v^{1/2}$

$$k = (2.69 \times 10^5)n^{3/2}AD_O^{1/2}C_O^* \qquad (5)$$

$$D_O^{1/2} = \frac{k}{(2.69 \times 10^5)n^{3/2}AC_O^*} \qquad (6)$$

Hence, the diffusion coefficient D_O is calculated as follows:

$$D_O = \left(\frac{k}{(2.69 \times 10^5)n^{3/2}AC_O^*}\right)^2 \qquad (7)$$

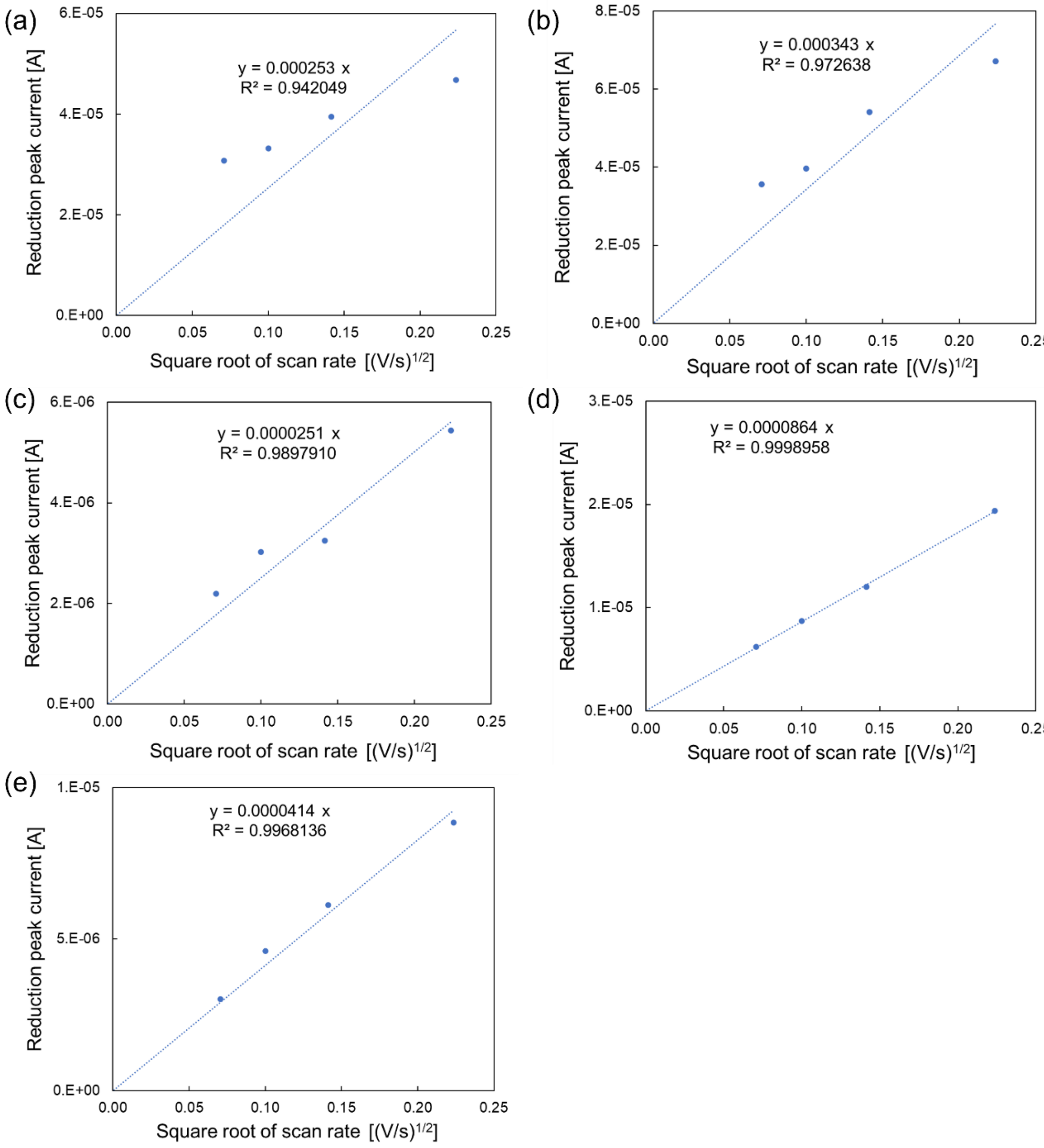

Figure S8. Plots of reduction peak current i_p^c versus the square root of the scan rate $v^{1/2}$ in Figure S7. k is the slope of linear approximation. (a) 1 mM Bu_4NClO_4 acetonitrile solution, (b) 10 mM Bu_4NClO_4 acetonitrile solution, (c) [DEME][BF_4], (d) [EMIM][BF_4] and (e) [DEME][TFSI].

AC-bipolar electropolymerization for 2 h at 10 V with 5 Hz (SQU)

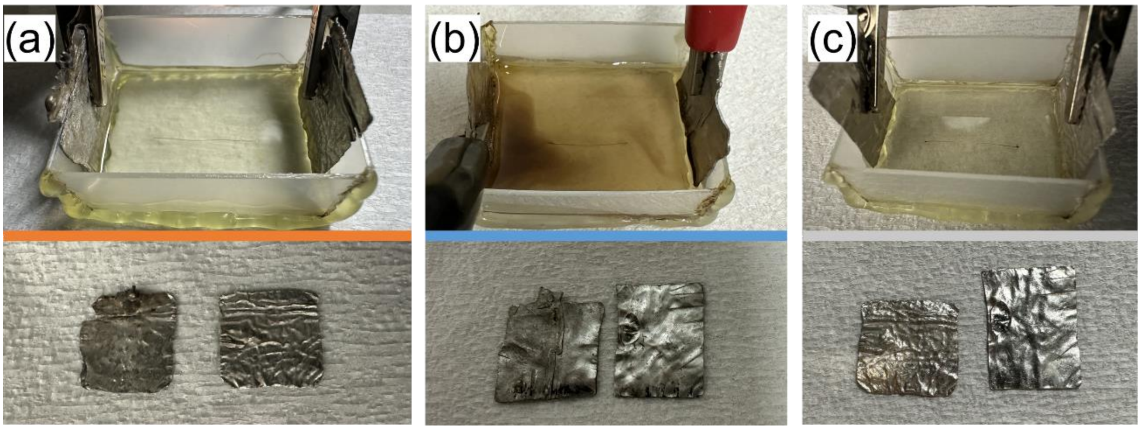

Figure S9. Photographs of electrolytic cells (upper) and the driving electrodes (bottom) after 2 h electrolysis at 10 V with 5 Hz (SQU). (a) [DEME][BF_4], (b) [EMIM][BF_4] and (c) [DEME][TFSI]. There was no polymer deposition on the driving electrodes in (a) and (c), whereas a thin film formed in (b).

AC-bipolar electropolymerization for 2 h at 10 V with 1 Hz and 50 Hz (SQU)

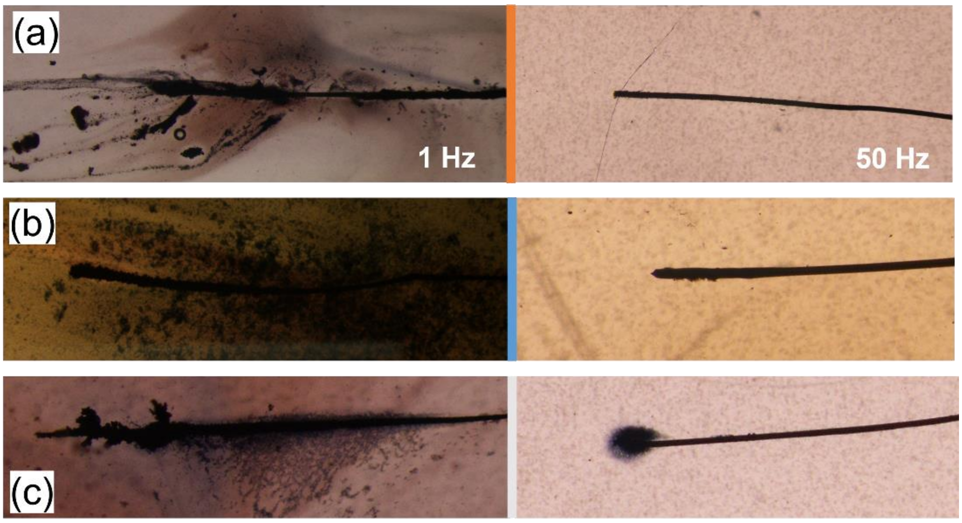

Figure S10. Optical microscope images of PEDOT obtained by AC-bipolar electropolymerization for 2 h at 10 V with 1 Hz (left) and 50 Hz (SQU) (right). (a) [DEME][BF_4], (b) [EMIM][BF_4], and (c) [DEME][TFSI].

AC-bipolar electropolymerization for 1 h at 20 V with 5 Hz (SQU)

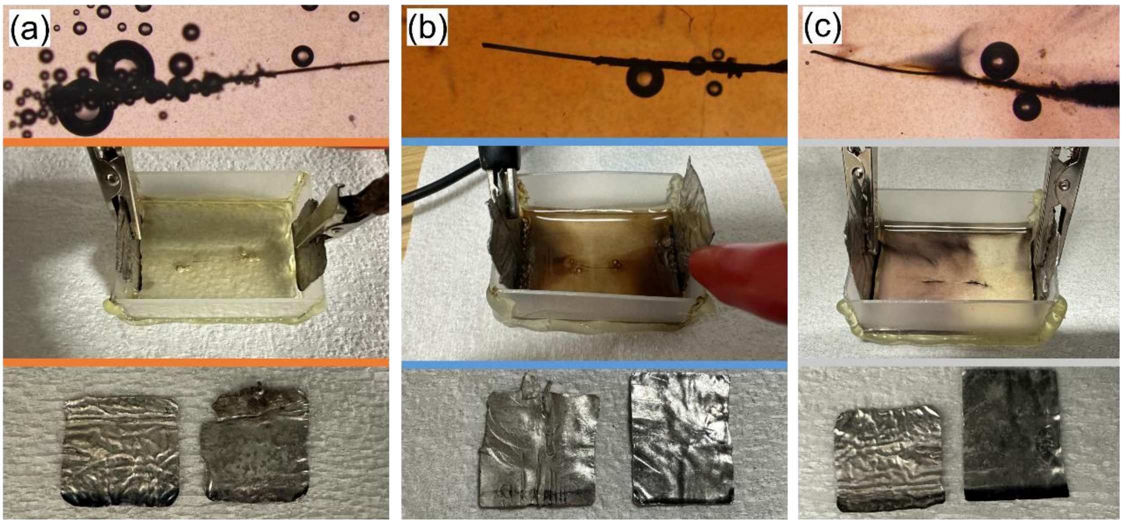

Figure S11. Optical microscope images of PEDOT obtained by AC-bipolar electropolymerization at 20 V with 5 Hz (SQU) (upper). The photographs of electrolytic cells (middle) and the driving electrodes (bottom) after electrolysis. (a) [DEME][BF_4], (b) [EMIM][BF_4], and (c) [DEME][TFSI].

References

1. Loget, G.; Roche, J.; Kuhn, A. True Bulk Synthesis of Janus Objects by Bipolar Electrochemistry. *Adv. Mater.* **2012**, *24*, 5111–5116.
2. Bard, A. J.; Faulkner, L. R. *Electrochemical Methods: Fundamentals and Applications*, 2nd ed., John Wiley & Sons, Inc., **2001**.
